# Viral and cellular requirements for the budding of Feline Endogenous Retrovirus RD-114

**DOI:** 10.1186/1743-422X-8-540

**Published:** 2011-12-14

**Authors:** Aiko Fukuma, Masumi Abe, Shuzo Urata, Rokusuke Yoshikawa, Yuko Morikawa, Takayuki Miyazawa, Jiro Yasuda

**Affiliations:** 1 Department of Emerging Infectious Diseases, Institute of Tropical Medicine, Nagasaki University, Nagasaki 852-8523, Japan; 2 Fifth Biology Section for Microbiology, First Department of Forensic Science, National Research Institute of Police Science, Kashiwa 277-0882, Japan; 3 Kitasato Institute for Life Sciences and Graduate School for Infection Control, Kitasato University, Tokyo 108-8641, Japan; 4 Laboratory of Signal Transduction, Institute for Virus Research, Kyoto University, Kyoto 606-8507, Japan

**Keywords:** RD-114, Endogenous retrovirus, Budding, WWP2, MVB sorting, Vaccine

## Abstract

**Background:**

RD-114 virus is a feline endogenous retrovirus and produced as infectious viruses in some feline cell lines. Recently, we reported the contamination of an infectious RD-114 virus in a proportion of live attenuated vaccines for dogs and cats. It is very difficult to completely knock out the RD-114 proviruses from cells, as endogenous retroviruses are usually integrated multiply into the host genome. However, it may be possible to reduce the risk of contamination of RD-114 virus by regulating the viral release from cells.

**Results:**

In this study, to understand the molecular mechanism of RD-114 virus budding, we attempted to identify the viral and cellular requirements for RD-114 virus budding. Analyses of RD-114 L-domain mutants showed that the PPPY sequence in the pp15 region of Gag plays a critical role in RD-114 virus release as viral L-domain. Furthermore, we investigated the cellular factors required for RD-114 virus budding. We demonstrated that RD-114 virus release was inhibited by overexpression of dominant negative mutants of Vps4A, Vps4B, and WWP2.

**Conclusions:**

These results strongly suggest that RD-114 budding utilizes the cellular multivesicular body sorting pathway similar to many other retroviruses.

## Background

The cat genome contains an infectious endogenous retrovirus (ERV), named RD-114 virus [1-3]. The RD-114 virus is a recombinant comprised of the *gag-pol *genes from a gammaretrovirus and the env gene from a betaretrovirus [4]. The amounts of viral RNA reach approximately 100 copies per cells in feline cells [3]. Some feline cell lines, such as Crandell-Rees feline kidney (CRFK) cells, constitutively express infectious RD-114 virus [5,6]. These cells have been used to grow several live attenuated vaccines for cats and dogs. Recently, we reported the isolation of an infectious RD-114 virus in a proportion of live attenuated vaccines for pets [7-9]. RD-114 is a polytropic virus and has potential risks in that interspecies transmission may induce unpredictable diseases, although the pathogenicity of RD-114 has not been demonstrated [5]. However, it is very difficult to completely exclude the proviral DNA of RD-114 from cells, as ERVs are usually integrated into multiple loci of the host chromosomes. Therefore, it may be a practical strategy to reduce the risk of contamination of RD-114 virus by regulating the release of infectious RD-114 from cells.

It is unknown how RD-114 virus buds from the plasma membrane of infected cells. Gag proteins of many retroviruses include short peptide motifs required for virus budding, i.e., L-domains. To date, three types of L-domain motif have been identified: PT/SAP, PPxY, and YxxL [10]. The PTAP motif interacts with Tsg101, an ubiquitin-conjugating E2 enzyme variant (UEV) and the PPxY motif interacts with Nedd4-like E3 ubiquitin ligases [11-16]. The cellular factor that interacts with the YxxL motif has been shown to be AIP1/Alix [17]. These host factors are the cellular proteins involved in the multivesicular body (MVB) sorting pathway. The MVB complex consists of a network of class E vacuolar protein sorting proteins, which form four distinct heteromeric endosomal sorting complexes required for transport known as ESCRT (endosomal sorting complexes required for transport)-0, -I, -II, and -III; these four complexes are required for the formation and release of the vesicles of the MVB [11,17-20]. Their major function in the cell is to transport cargo proteins, such as activated cell surface receptors, from the early endosomal membrane to be released into the lysosome in small vesicles for degradation. The proteins are targeted to the endosomal degradation pathway by modification with mono- to tetraubiquitin chains. The components of ESCRT complexes participate in inward invagination and budding of late endosomal membranes to form MVB. Therefore, the processes of virus budding and MVB vesicle budding are considered fundamentally the same, although they occur at different sites in the cell. The ESCRT machinery is processed by sequential interaction of target protein with ESCRT-0, -I, -II, and -III [21]. Vps4 is an AAA ATPase and performs a key function in this pathway by recognizing membrane-associated ESCRT-III assemblies and catalyzing their disassembly, possibly in conjunction with membrane fission. It has been reported that abrogations of the functions of these cellular factors using dominant-negative mutants or siRNA inhibit viral release of various enveloped viruses possessing L-domains.

In this study, to obtain the information useful for the inhibition of RD114 release from cells, we analyzed the molecular mechanism of RD-114 virus budding.

## Results

### The PPPY sequence functions as a major L-domain in RD-114 replication

**Figure 1:**
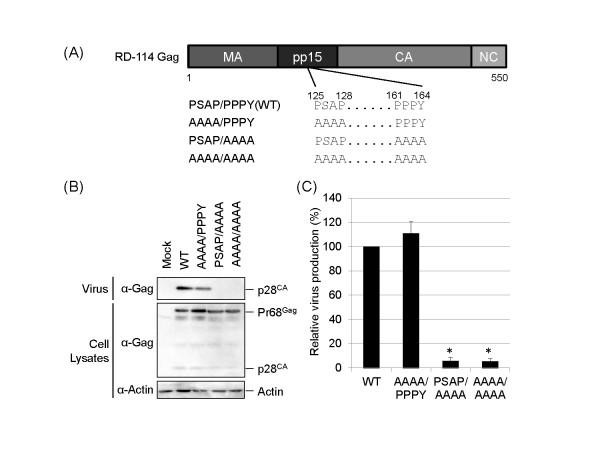
**Identification of the L-domain, which is critical for RD-114 virus budding**. (**A**) Schematic representation of RD-114 Gag and the L-domain mutants. The positions of the two putative L-domain motifs are indicated. (**B **and **C**) 293 T cells were transfected with the RD-114 infectious clone for wild-type (pTERD-114) or the L-domain mutant (0.5 μg). Cells and viruses were collected at 72 h after transfection, and analyzed by Western blotting (**B**) and real-time RT-PCR targeting the *pol *region (**C**). The virus production from cells expressing wild-type (WT) RD-114 was set to 100%. The data are shown as averages and standard deviations of 3 independent experiments. Statistical analysis was performed by using the Student's *t*-test (*: *p *< 0.01)

To examine the functions of two putative L-domain motifs in the pp15 region of RD-114 Gag, PSAP and PPPY, we constructed expression plasmids for the RD-114 mutants, which substituted PSAP, PPPY, or both sequences to all alanine, AAAA (Figure 1A). The expression plasmids for wild-type (WT) or mutant RD-114 were transfected into 293 T cells. At 72 h posttransfection, the amounts of the intracellular Gag precursor (Pr68) and the capsid protein (p28CA) in virus particles were analyzed by Western blotting using anti-RD-114 Gag antibodies. As shown in Figure 1B, similar levels of Gag precursor were synthesized in cells expressing either WT or mutant genomes. The release of virus particles into the medium was greatly reduced by alanine substitution of the PPPY motif, while the PSAP mutation had no apparent effect on virus production. Moreover, the AAAA/AAAA mutant with the mutation introduced into both L-domain motifs, PSAP and PPPY, showed the reduction of virus release as well as the PSAP/AAAA mutant. The levels of virus production of mutants were also quantitatively analyzed by real-time RT-PCR targeting the *pol *region (Figure 1C). The progeny virus production of the PSAP/AAAA mutant or the AAAA/AAAA mutant was only 5.9% or 5.5% of that of WT, respectively (*p *< 0.01), while the AAAA/PPPY mutant produced progeny virion at the similar level to WT. Thus, the results of real-time RT-PCR were consistent with those of Western blotting analysis. These data suggest that the PPPY motif, but not the PSAP motif, plays a major role as an L-domain in RD-114 budding.

### RD-114 production is inhibited by overexpression of the dominant-negative mutant of WWP2

**Figure 2:**
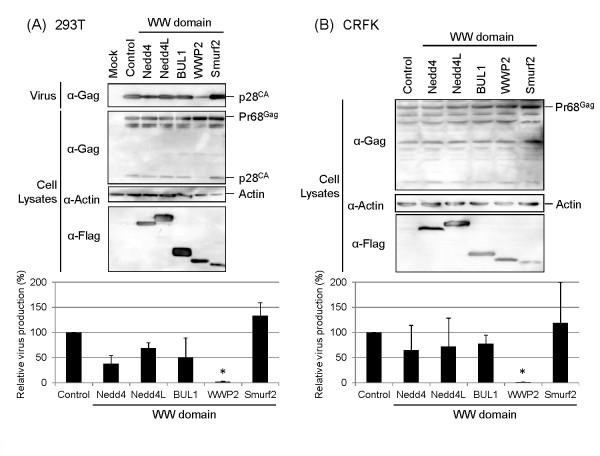
**Effects of dominant-negative mutants of Nedd4-like E3 ubiquitin ligases on RD-114 production**. (**A**) 293 T cells were cotransfected with pTERD-114 (100 ng) and either the expression plasmid for the dominant-negative mutants of Nedd4-like E3 ubiquitin ligases, which express only WW domains containing N-terminal FLAG-tag, or the empty vector pCDNFL (Control) (1 μg). Cells and viruses were collected at 72 h after transfection, and analyzed by Western blotting (upper) and real-time RT-PCR (lower). The virus production in Control was set to 100%. The data are shown as averages and standard deviations of 3 independent experiments. (**B**) CRFK cells were transfected with the expression plasmid for the dominant-negative mutant of Nedd4-like E3 ubiquitin ligases (1 μg) and analyzed as in (**A**). Statistical analysis was performed by using the Student's *t*-test (*: *p *< 0.01)

It has been reported that the PPxY sequence identified as a viral L-domain in various viruses interacts with the WW domains of the cellular Nedd4-like E3 ubiquitin ligases [13,15]. This interaction is essential for the function of the PPxY L-domain motif in virus budding, although the precise role of Nedd4-like E3 ubiquitin ligases in virus budding has not been clarified. To examine the involvement of Nedd4-like E3 ubiquitin ligases in the egress of RD-114, the RD-114 infectious clone, pTERD-114, was cotransfected with plasmids directing expression of the dominant-negative mutants of Nedd4, Nedd4L, BUL1, WWP2, and Smurf2, Nedd4-WW, Nedd4L-WW, BUL1-WW, WWP2-WW, and Smurf2-WW, which express only WW domains, into 293 T cells and monitored for impact on RD-114 virus production by Western blotting and real-time RT-PCR as in Figure 1. As shown in Figure 2A, virus production was significantly reduced to 2% of control by overexpression of WWP2-WW (*p *< 0.01). The effects of these WW domains of the cellular Nedd4-like E3 ubiquitin ligases were also investigated in CRFK cells, which is a feline cell line constitutively expressing infectious endogenous RD-114. The analysis was performed by real-time RT-PCR as it was difficult to detect the low levels of RD-114 virus production from CRFK cells by Western blotting (Figure 2B). It was shown that WWP2-WW also significantly inhibited the production of endogenous RD-114 expression to 1% of control in CRFK cells. These results suggest that WWP2 or another HECT E3 ubiquitin ligase closely related to WWP2 is involved in RD-114 budding.

### RD-114 budding depends on ESCRT machinery

**Figure 3:**
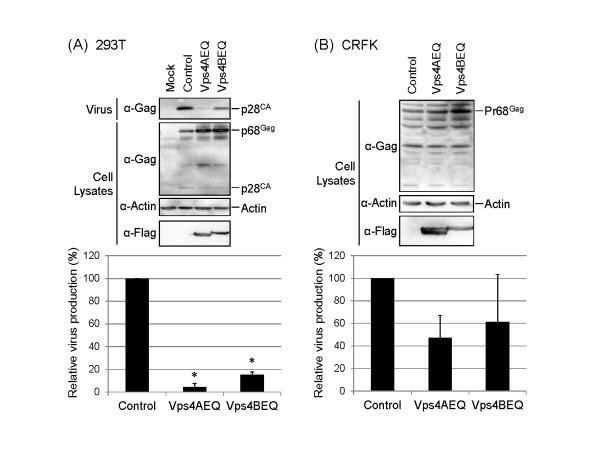
**Inhibition of RD-114 production by overexpression of the dominant-negative mutants of Vps4A/B**. (**A**) 293 T cells were cotransfected with pTERD-114 (100 ng) and either the expression plasmids for Vps4A/B dominant-negative mutant containing N-terminal FLAG-tag, or the empty vector pCDNFL (Control) (100 ng), and then analyzed by Western blotting (upper) and real-time RT-PCR (lower) as in Figure 1. The virus production in Control was set to 100%. The data are shown as averages and standard deviations of 3 independent experiments. Statistical analysis was performed by using the Student's *t*-test (*: *p *< 0.01). (**B**) CRFK cells were transfected with the expression plasmids Vps4A/B dominant-negative mutant (1 μg). Cells and viruses were collected at 72 h after transfection, and analyzed by Western blotting (upper) and real-time RT-PCR (lower) as in (**A**).

Identification of functional L-domain in RD-114 Gag and the involvement of Nedd4-like E3 ubiquitin ligase in RD-114 budding strongly suggest that RD-114 utilizes the ESCRT machinery in its budding similar to many other enveloped viruses [10]. It has been reported that overexpression of the ATPase-deficient mutant of Vps4A or Vps4B, Vps4AE228Q or Vps4BE235Q, inhibited the release of a unique subset of ESCRT-utilizing retroviruses depending on L-domain differences in a dominant-negative manner [11]. To determine whether RD-114 virus budding utilizes the ESCRT machinery, 293 T cells were cotransfected with the plasmid for Vps4AE228Q or Vps4BE235Q along with pTERD-114. At 72 h posttransfection, viral protein synthesis and virion release were analyzed by Western blotting and real-time RT-PCR as shown in Figure 1. We observed that virus release was significantly reduced to 4.4% or 15%, respectively, by the overexpression of Vps4AEQ or Vps4BEQ (*p *< 0.01) (Figure 3A). We also analyzed the effects of Vps4AE228Q or Vps4BE235Q overexpression in CRFK cells (Figure 3B). The virus release of RD-114 endogenously expressed in CRFK cells was also inhibited by the overexpression of Vps4AE228Q or Vps4BE235Q, indicating that RD-114 utilizes the cellular ESCRT machinery in the budding of progeny viruses.

## Discussion

In this study, we demonstrated that the PPPY sequence in the pp15 region of RD-114 Gag plays a critical role in virus production as a major L-domain and that WWP2 or WWP2-like E3 ubiquitin ligases possessing the WW domain closely related to WWP2 and Vps4A/B are involved in RD-114 budding. These data suggest that RD-114 Gag recruit the cellular ESCRT machinery through the interaction of the PPPY L-domain with the WW domain(s) of WWP2 and progeny virions are released from cells by utilizing the MVB sorting pathway similar to many other retroviruses. Therefore, strategies targeting these cellular proteins might be effective to suppress endogenous RD-114 production from feline cells. In fact, overexpression of dominant-negative mutants of WWP2 and Vps4A/B could markedly inhibit the production of endogenous RD-114 viruses from CRFK cells as well as exogenous RD-114 viruses from 293 T cells (Figures 2 and 3).

WWP2 is known to negatively regulate transcriptional activity of Oct-4, which is a transcription factor critical in mammalian embryonic development, by promoting ubiquitination of Oct-4 [22]. Oct-4 is present in cultured undifferentiated embryonic cell lines, including embryonic stem (ES) cells, embryonal carcinoma cells, and embryonic germ cells, while it is absent from all of the differentiated somatic cell types *in vitro *and *in vivo *[23]. A recent study also showed that WWP2 inhibits activation-induced T-cell death by catalyzing ubiquitination of EGR2, which is a zinc finger transcription factor that regulates Fas ligand (FasL) expression [24]. Therefore, abrogation of WWP2 function by constitutive expression of dominant-negative mutant may be possible in most of the differentiated somatic cell lines that have been used for production of vaccine or biological substances.

Furthermore, we recently reported that matrix proteins (VP40) of Ebola and Marburg viruses recognize a different WW domain within Nedd4.1, although both VP40 proteins interact with Nedd4.1 via their PPxY L-domain [25]. WWP2 contains four WW domains [22]. Identification of the WW domain that specifically binds to RD-114 Gag may enable the development of strategies that have minimal effect on physiological function of WWP2 and efficiently reduce endogenous RD-114 production.

Strategies targeting Vps4 might also be available to reduce endogenous RD-114 production. Recently, a stable cell line with inducible expression of a dominant-negative form of Vps4 has been established [26]. This method would be applicable to the various cellular factors the dominant-negative forms of which inhibit RD-114 production.

Taken together, our data would be useful for development of the strategies to control virus production in cells constitutively producing infectious endogenous viruses.

## Conclusions

In this study, we revealed that the PPPY sequence in the pp15 region of Gag plays a critical role in the virus budding of RD-114 as viral L-domain. Furthermore, we demonstrated that RD-114 virus production was significantly inhibited by overexpression of dominant-negative mutants of Vps4A, Vps4B, and WWP2. These results strongly suggest that RD-114 budding utilizes the MVB sorting pathway similar to many other retroviruses.

## Methods

### Cells

Human embryonic kidney (HEK) 293 T cells (ATCC CRL-11268), and Crandell-Rees feline kidney (CRFK) cells (ATCC CCL-94) were maintained at 37°C in a 5% CO_2 _incubator in Dulbecco's modified Eagle's medium (Sigma, St. Louis, MO) supplemented with 10% fetal bovine serum and penicillin/streptomycin.

### Plasmids

A plasmid containing an intact infectious clone of RD-114, pTERD-114, was constructed from TE/RD114 cell [27,28]. To generate the expression plasmids for the RD-114 L-domain mutants, AAAA/PPPY, PSAP/AAAA, and AAAA/AAAA (Figure 1A), alanine substitutions were introduced into pTERD-114 by site-directed mutagenesis using a KOD-Plus-Mutagenesis Kit (Toyobo, Osaka, Japan). The expression plasmids for each dominant-negative mutant of Nedd4, BUL1, Vps4A, and Vps4B: pNedd4-WW (NP_006145, amino acid region 192-506), pBUL1-WW (NP_055867, amino acid region 830-1051), pVps4AE228Q, and pVps4BE235Q, respectively, were constructed previously [15,16,29]. The expression plasmids for each dominant-negative mutant of Nedd4L, WWP2, and Smurf2: pNedd4L-WW (NP_001138442, amino acid region 68-456), pWWP2-WW (NP_008945, amino acid region 293-485), and pSmurf2-WW (NP_073576, amino acid region 151-338), were constructed in this study. Each WW construct of Nedd4L, WWP2, and Smurf2 was amplified by standard PCR using primer containing KpnI sites from the human spleen Marathon-Ready cDNA library (Clontech, Mountain View, CA). These products were subcloned into pCDNFL, which was constructed from pcDNA3.1 (Invitrogen, Carlsbad, CA) to express a protein containing a FLAG-tag at the N terminus.

### Production of anti-RD-114 Gag antibody

RD-114 Gag (amino acid residues 225-550) was expressed in Escherichia coli BL21 as a GST fusion protein (from pGEX6P-2; GE Healthcare, Piscataway, NJ), and purified from the bacterial extracts according to the manufacturer's instructions. As the GST fusion proteins attached to the glutathione-Sepharose 4B beads were digested with PreScission protease (GE Healthcare), the purified Gag protein did not contain GST. The purified Gag protein was used to immunize rabbits.

### Western blot analyses

For L-domain mutant analysis, 293 T cells (2 × 10^5^) were transfected with the expression plasmid for RD-114 wild-type or L-domain mutant, AAAA/PPPY, PSAP/AAAA, or AAAA/AAAA, using Trans-IT LT-1 (Mirus Bio Corp., Madison, WI). For analyses of the cellular factors, 293 T or CRFK cells (2 × 10^5^) were cotransfected with pTERD-114 (100 ng) and pNedd4-WW, pNedd4L-WW, pBUL1-WW, pWWP2-WW, pSmurf2-WW, pVps4AE228Q, or pVps4BE235Q (0.1 or 1 μg) using Trans-IT LT-1. At 72 h after transfection, the supernatants were separated from cell debris by centrifugation (10,000 × g; 15 min) and then virions were pelleted through a 16.5% sucrose cushion by ultracentrifugation (348,000 × g; 40 min at 4°C) [27]. Pelleted virions were resuspended in PBS(-). Cells were lysed with lysis A buffer [30]. Cell lysates and virions were analyzed by Western blotting using anti-RD-114 Gag antibody, anti-FLAG M2 antibody (Sigma), and anti-actin antibody (Sigma) as described previously [27,29,31,32].

### Real-time RT-PCR

To quantify the release of progeny virions from cells, virions were prepared as described in Western blot analyses. Viral RNAs were extracted from pelleted virions using a QIAamp Viral RNA Mini Kit (QIAGEN, Valencia, CA). After DNaseI treatment, real-time RT-PCR was performed using a One Step SYBR RT-PCR Kit (Takara, Shiga, Japan). The primers targeting the RD-114 *pol *region, 5'-GAGACCCTTACTAAATTGAC-3' (forward) and 5'-AGTTTCTGGTCCAGGGGTTT-3' (reverse), were used for real-time RT-PCR [33]. The amplification was carried out in a 25-μl reaction volume and contained 12.5 μl of 2 × One Step SYBR RT-PCR Buffer III, 2.5 U of TaKaRa Ex Taq HS, 0.5 μl of PrimeScript RT enzyme Mix II, 5 pmol of forward and reverse primers, and 2 μl of RNA sample. The thermal profile was at 42°C for 5 min and 95°C for 10 s, followed by 45 cycles of 95°C for 5 s, 60°C for 20 s, and 72°C for 15 s. Thermal cycling and quantification were performed using a Smart Cycler II System (Cepheid, Sunnyvale, CA).

## Abbreviations

ERV: endogenous retrovirus; CRFK: Crandell-Rees feline kidney; UEV: ubiquitin-conjugating E2 enzyme variant; ESCRT: endosomal sorting complexes required for transport; MVB: multivesicular body; WT: wild-type; ES cells: embryonic stem cells; FasL: Fas ligand; HEK 293 T cells: Human embryonic kidney 293 T cells.

## Competing interests

The authors declare that they have no competing interests.

## Authors' contributions

AF designed the study, carried out experiments, participated in analysis of the results, and wrote the manuscript. MA and SU participated in analysis of the results. YM helped in drafting the manuscript and performed critical revisions. TM designed the study and participated in analysis of the results. JY designed the study, participated in analysis of the results, and participated in manuscript preparation. All authors have read and approved the final manuscript.
